# Method for verifying the air kerma strength of I‐125 plaques for the treatment of ocular melanoma

**DOI:** 10.1120/jacmp.v15i4.4880

**Published:** 2014-07-08

**Authors:** L. W. Zimmermann, D. Allan Wilkinson

**Affiliations:** ^1^ Department of Physics Cleveland State University Cleveland OH USA; ^2^ Department of Radiation Oncology Cleveland Clinic Cleveland OH USA

**Keywords:** brachytherapy, ocular melanoma, Iodine‐125, I‐125, eye plaque

## Abstract

The purpose of this work was to develop a method for easily verifying that the activity or air kerma strength of pre‐assembled eye plaques, used in the treatment of ocular melanomas, agrees with the activity or air kerma strength called for in the treatment plan. A Capintec CRC‐7 Dose Calibrator with its standard vial/syringe sample holder was used to measure the activity of pre‐assembled COMS and Eye Physics EP917 eye plaques using IsoAid Advantage I‐125 seeds. Plaque activity measurements were made by placing the plaque face up in the center of a 5 cm tall Styrofoam insert in the source holder. Activity measurements were made with the source holder rotated to four angles (0°, 90°, 180°, and 270°). The average of these four values was converted to air kerma strength and divided by the assay air kerma strength, from the NIST traceable source calibration, and decayed to the plaque measurement date, to determine a plaque calibration factor. The average of the calibration factors for each plaque type was used to establish a calibration factor for each plaque type. Several partially loaded plaque configurations were included in this study and different methods were used to determine the effects of partial loading. This verification method is easy to implement with commonly available equipment and is effective in identifying possible errors. During this two‐year study, the air kerma strength of 115 eye plaques was checked and 11 possible errors were identified.

PACS number: 87.55.Qr

## I. INTRODUCTION

The most common radiation treatment for ocular melanomas is episcleral plaque brachytherapy in which a small eye plaque containing radioactive seeds (usually Iodine‐125 and Palladium‐103 in the USA) is sutured to the scleral surface over the tumor base. The plaque is left in place for a few days and then removed. In comparison to enucleation (removal of eye), plaque brachytherapy offers equivalent tumor control while allowing eye preservation and the possibility of vision retention.^(1)^

As with other forms of brachytherapy, quality assurance measures are needed to minimize the risk of treatment delivery errors. For plaques that are to be assembled in‐house, it is good practice, as well as mandated by regulation, that at least 10% of the seeds be assayed to confirm the source strength. Third‐party source handling and calibration services may be substituted and may, in fact, provide an assay of all the sources in a customer's order. Our practice is to assay at least 25% and usually 100% of the sources prior to loading them into eye plaques. However, if the plaque comes already assembled, another method for checking that the correct source strength has been provided needs to be in place. In addition to calibration mistakes, errors unrelated to the assay may remain undetected. These errors can occur during placing or filling of seed orders and can include confusing the units of source strength (air kerma strength versus apparent activity in millicuries^(2)^), transcription errors, wrong patient data, and shipment of a wrong plaque.

The American Brachytherapy Society Recommendations for Brachytherapy of Uveal Melanomas^(3)^ does not specify any quantitative dosimetry verification for pre‐assembled eye plaques. Visual inspections of the assembled plaque through a lead glass barrier or a radiograph of the loaded seed carrier or pre‐assembled plaque have been the only options for quality assurance of the assembled plaques. These methods are adequate for verifying that the plaque is loaded according to the planned loading pattern, but they cannot verify the activity or strength of the seeds.

Other methods have been used, such as high‐spatial resolution 2D and 3D dosimetry using a silicon pixelated detector^4^, ^5^ and a pinhole camera combined with a computed radiography CR unit to image the location and measure the relative strength of the seeds and a survey meter to estimate the total activity of the seeds in the plaque.^(6)^

More recently, AAPM Task Group 129^(7)^ was formed to: a) review the dosimetry aspects of eye plaque brachytherapy, b) evaluate the impact of implementing the recommendations of TG‐43U1 for the homogeneous assumption, c) examine the heterogeneity effects on the dose distributions in the eye tumor and critical ocular structures, and d) make recommendations for treatment planning and quality assurance (QA) for eye‐plaque brachytherapy. The report of Task Group 129 recommends the following quality assurance process for pre‐assembled eye plaques: “A recent development has been the availability of prepared plaques for rent from source manufacturers. These plaques come sterilized with no opportunity for direct measurement of source strength. A minimum of one nonsterile loose seed should be ordered and assayed by the in‐house physicist, given that the number of seeds ranges from 5 to 24 in fully loaded 10 mm–22 mm COMS plaques. The assay tolerance for the air kerma strength of the loose seed(s) is 6% individually and 3% for any single batch.” This is similar to the recommendations in TG‐40 and TG‐56 reports for calibration of a single loose source from each strength grouping when the remaining sources intended for implantation were in sterile strands or assemblies.^(8)^ Of course, the validity of this type of check rests upon the assumption that the supplied source is representative of those in the plaque. Each preloaded plaque, as well as all seed orders from IsoAid, come with a 100% NIST‐traceable third‐party calibration.

We have investigated a method for performing dosimetric verification of loaded eye plaques prior to use; this applies to plaques ordered as pre‐assembled, as well as in‐house‐assembled plaques. This paper describes this method of verifying the activity or air kerma strength of COMS and Eye Physics EP917 eye plaques.

COMS (Collaborative Ocular Melanoma Study) plaques consist of a gold alloy, concave, semispherical shell with a radius of curvature designed to conform to the scleral surface. A small lip around the outer edge of the shell retains a Silastic seed carrier insert, which contains the brachytherapy seeds. COMS plaques are available in diameters from 10 to 22 mm in 2 mm increments. The Eye Physics, LLC (Los Alamitos, CA) EP917 eye plaque is a 16×14 mm, semielliptical gold alloy plaque with a radius of curvature designed to conform to the scleral surface. There are 17 0.8 mm deep slots in the plaque that hold the brachytherapy seeds, which are glued in place.

In addition to having measured fully loaded symmetric plaques, we measured notched COMS plaques and partially loaded COMS plaques and EP917s to determine how the plaque calibration factor is affected by the asymmetry of the partially loaded plaques. Since the seeds in the Eye Physics EP917 plaques are not laid out in a uniform pattern and are placed in slots within the gold plaque, it was suspected that the activity measurements for plaques that are not fully loaded would differ from those that were fully loaded. Since the seeds in this plaque are placed in slots, the collimation of each source reduces the laterally directed primary radiation measured by the dose calibrator.

## II. MATERIALS AND METHODS

**Figure 1:**
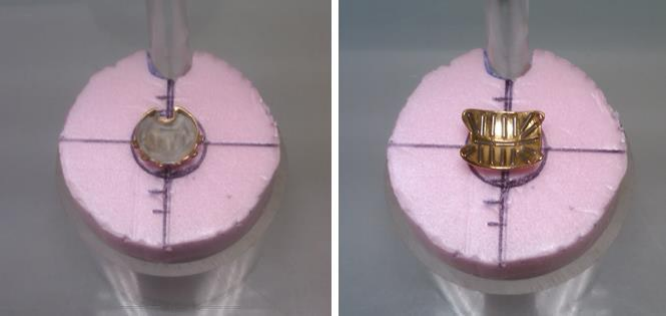
COMS plaque (left) and EP917 (right) centered on Styrofoam block in dose calibrator source holder. Crosshairs were drawn on the Styrofoam blocks to facilitate centering of the plaques. Plaques were held in place by double‐sided adhesive tape.

A Capintec CRC‐7 Dose Calibrator (Capintec, Inc., Ramsey, NJ) with its standard sample holder was used to measure the activity of pre‐assembled eye plaques loaded with IsoAid Advantage I‐125 seeds (IsoAid LLC, Port Richey, FL). The dose calibrator constancy was verified by use of a ${\;}^{137}\text{Cs}$ vial source prior to each plaque measurement. Plaque activity measurements were made by placing the plaque face up in the center of a 5 cm tall Styrofoam insert in the sample holder, as shown in Fig. 1. The 5 cm tall Styrofoam insert was used to raise the plaque above the top of the plastic sample holder to make it easier to position. Double‐sided tape was used to keep the plaque from moving while the sample holder was rotated during the measurement process. Centering the plaque was facilitated by marking a crosshair on the insert.

The sample holder was rotated to four angles (0°, 90°, 180°, and 270°) and the activity recorded. The four measurements at four angles of rotation are used to minimize any geometry differences in plaque placement in the sample holder. The difference between the four measurements taken at the four angles of rotation can be made quite small (typically less than 4%) by careful centering of the plaque. The plaque orientation shown in Fig. 1 was used for all activity measurements in this study to reproducibly position the plaques on the Styrofoam insert. In the event that the deviation between any of the four readings was larger than 4%, the plaque was repositioned and the four measurements repeated. The average of these four activity values was converted to air kerma strength (1U=0.787 mCi for ${\;}^{125}I$), and compared to the assay air kerma strength (third‐party NIST‐traceable calibration), decayed to the plaque measurement date, to determine a plaque calibration factor.

A spreadsheet was used to record the plan and assay seed data (air kerma strength and calibration date) and measured plaque activity. The spreadsheet then calculated the air kerma strength of the seeds decayed to the plaque measurement date. Finally, the plaque calibration factor was calculated by dividing the measured air kerma strength by the decayed assay strength. A sample showing the results for the first ten EP917 eye plaques is shown in Table 1.

**Table 1:** Plaque calibration factor worksheet showing assay data, measured plaque data, and plaque calibration factor comparison results

	*Assay Data*			*Measured Data*			*Measured vs. Assay*		
*Plaque ID*	*No. of Seeds*	*Assay Date*	*Air Kerma Strength*	*Date*	*Activity*	*Air Kerma Strength*	*Decayed Strength*	*Cal Factor*	*Deviation from Mean*
1	17	9/16/2011	115.60	10/23/2011	20.30	25.78	75.07	0.343	−2.8%
2	17	12/2/2011	51.19	12/8/2011	14.05	17.84	47.73	0.374	5.9%^a^
3	15	12/2/2011	40.62	12/8/2011	10.58	13.43	37.87	0.355	0.4%
4	15	12/2/2011	36.86	12/8/2011	9.24	11.73	34.37	0.341	−3.3%
5	6	12/2/2011	43.18	12/8/2011	11.19	14.21	40.26	0.353	0.0%
6	17	1/6/2012	65.35	1/10/2012	16.90	21.46	62.37	0.344	−2.6%
7	17	1/20/2012	70.94	1/23/2012	18.21	23.12	68.50	0.338	−4.4%
8	17	3/9/2012	59.13	3/8/2012	17.23	21.88	59.82	0.366	3.6%
9	17	3/23/2012	53.55	3/22/2012	15.57	19.78	54.18	0.365	3.4%
10	17	4/2/2012	47.52	3/30/2012	13.10	16.64	49.21	0.338	−4.3%

a Plaque calibration factor that deviate by more than ±5% from the mean calibration factor for the plaque type.

The calibration factors for each type of plaque were averaged to establish a calibration factor for each plaque type, and the deviation from this average was calculated for each plaque. Plaques with calibration factors exceeding ±5% from the average for the plaque type are highlighted, as they may require further investigation. The standard deviation of the plaque calibration factors for each plaque type was used as a measure of the repeatability of the process.

The 5% action limit is based upon the recommendations of the AAPM low energy Brachytherapy Source Calibration Working Group.^(8)^ In the AAPM report, Table II describes the actions to be taken by the physicist at the end‐using institution, based on the sample size assayed and the relative difference, ΔSK, found between the manufacturer's source strength certificate and the assay by the physicist at the using institution. From the AAPM report's Table II, an action limit of ΔSK>5% is used for a batch measurements of individual sterile strands, cartridges or preloaded needles. For this study, the activity measurement of a preloaded eye plaque is considered similar to the batch measurements of individual sterile strands, cartridges or preloaded needles.

In addition to checking each plaque against its assay data, several other quality assurance checks are performed; the entered plan and assay seed data are compared, and plaque calibration factors based upon the plan and assay data are compared. Variations in the seed data and plaque calibration factors that exceed ±5% are highlighted in red for easy identification.

Activity measurements were also made to determine the contribution that a single seed makes to the total activity of a fully loaded EP917 plaque. Two methods were used for these measurements: measuring the activity of a single seed placed in each of the seed positions, and removal of one seed at a time from a loaded plaque and calculation of the difference to the measured activities.

## III. RESULTS

### A. Results for preassembled Eye Physics EP‐917 plaques

**Figure 2:**
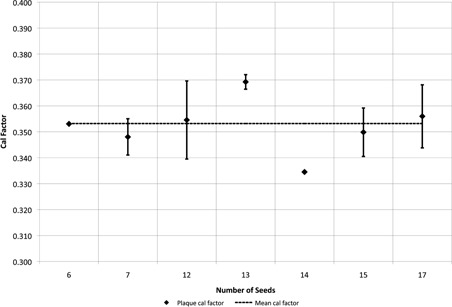
Plaque calibration factors for partially loaded Eye Physics EP917 plaques. The calibration factors for the partially loaded plaques are consistent with the mean value for all EP917 plaques of 0.353.

Activity measurements were taken on 56 vendor‐preloaded Eye Physics EP917 eye plaques (23 fully loaded; 33 partially loaded). For the EP917, the average plaque calibration factor was 0.353, with a coefficient of variation of 3.29%. The coefficient of variation (CV) is defined as the ratio of the standard deviation to the mean, expressed as a percentage. The partially loaded plaques were grouped by their number of seeds and calibration factors were calculated for each group. The calibration factors for the groups of partially loaded plaques were consistent with those for the fully loaded plaques. The calibration factors for the groups of partially loaded EP917 plaques are shown in Fig. 2 and in Table 2.

**Table 2:** Plaque calibration factors for partially loaded plaques

*Plaque Type*	*Number of Seeds*	*Plaque Cal Factor*	*Deviation From Mean Calibration Factor*	*Number of Plaques*
EP917	6	0.353	0.0%	1
EP917	7	0.348	−1.5%	2
EP917	12	0.355	0.4%	5
EP917	13	0.369	4.5%	2
EP917	14	0.335	−5.3%	1
EP917	15	0.350	−0.9%	22
COMS14N	12	0.254	0.0%	7
COMS14N	8	0.248	−2.5%	1
COMS14N	7	0.261	2.7%	1
COMS18	21	0.251	0.6%	2
COMS18	20	0.247	−1.3%	1

Individual seed measurements were made using three different EP917 plaques to assess the contribution of each seed to the total activity. The data from these measurements show that the contribution of a single seed to the total plaque activity is approximately 5.9% or 1/17th of the total activity; this is shown in Table 3. Each seed in the EP917 plaque contributes equally (approximately 1/17th of the total activity) to the total air kerma strength of the loaded plaque. Thus, the plaque calibration factor remains valid for partially loaded plaques.

**Table 3:** Table showing the individual seed contribution to the total activity of an EP917 plaque. Using the average data from three plaques, the contribution of a single seed is 1/17th of the total activity, regardless of its position in the plaque

	*Plaque #1*		*Plaque #2*		*Plaque #3*		*Average of Three Plaques*	
*Seed Position*	*Activity (mCi)*	*% of Total Activity*	*Activity (mCi)*	*% of Total Activity*	*Activity (mCi)*	*% of Total Activity*	*Average % of Total Activity*	*Deviation from Average*
1	1.340	5.6%	0.813	6.0%	0.925	5.5%	5.7%	−0.2%
2	1.590	6.7%	0.754	5.6%	1.065	6.3%	6.2%	0.3%
3	1.540	6.5%	0.784	5.8%	0.920	5.4%	5.9%	0.0%
4	1.520	6.4%	0.628	4.7%	1.125	6.7%	5.9%	0.0%
5	1.270	5.3%	0.878	6.5%	1.150	6.8%	6.2%	0.3%
6	1.300	5.5%	0.820	6.1%	0.825	4.9%	5.5%	−0.4%
7	1.240	5.2%	0.790	5.9%	1.000	5.9%	5.7%	−0.2%
8	1.210	5.1%	0.835	6.2%	1.013	6.0%	5.8%	−0.1%
9	1.290	5.4%	0.920	6.8%	0.885	5.2%	5.8%	−0.1%
10	1.310	5.5%	0.770	5.7%	0.905	5.4%	5.5%	−0.4%
11	1.220	5.1%	0.765	5.7%	1.115	6.6%	5.8%	−0.1%
12	1.410	5.9%	0.708	5.3%	0.765	4.5%	5.2%	−0.6%
13	1.400	5.9%	0.785	5.8%	1.120	6.6%	6.1%	0.2%
14	1.510	6.3%	0.840	6.2%	0.943	5.6%	6.1%	0.2%
15	1.610	6.8%	0.780	5.8%	0.970	5.7%	6.1%	0.2%
16	1.450	6.1%	0.803	6.0%	1.045	6.2%	6.1%	0.2%
17	1.600	6.7%	0.780	5.8%	1.130	6.7%	6.4%	0.5%
Totals	23.81		13.45		16.9			
					Average			5.9%
					Standard Deviation			0.3%

### B. Results for in‐house‐assembled COMS plaques

Plaque calibration factors were calculated for 11 different types of COMS plaques. The calculated plaque calibration factors ranged from 0.234 for a COMS 20 to 0.264 for a COMS 10. Generally, the plaque calibration factors for COMS plaques are similar; the mean of the calibration factors for all COMS plaques is 0.251, with a coefficient of variation of 4.8%. For the individual plaque types, the calibration factors vary from the mean by −6.5% for a COMS 20 to 5.5% for a COMS 10.

More accurate results can be realized by grouping the COMS plaque calibration factors by plaque type. Using this method, the COMS plaque calibration factors can be determined with an average coefficient of variation of 2.7%; these results are shown in Table 4.

A small number of partially loaded COMS plaques were included in this study, two partially loaded COMS14N plaques and a single COMS18. All of the remaining COMS plaques (56 plaques) were fully loaded. The calibration factors for the partially loaded COMS14N plaques were within −2.5% and +2.7% of the mean calibration factor for all COMS14N plaques, and the partially loaded COMS18 was within 1.3% of the mean calibration factor for all COMS18 plaques. A summary of the calibration factors for the partially loaded COMS plaques are shown in Table 2.

**Table 4:** Plaque calibration factors for 11 different types of COMS plaques

*Plaque Type*	*Plaque Cal Factor*	*Standard Deviation*	*Coefficient of Variation*	*Number of Plaques*
COMS10	0.264	0.007	2.6%	8
COMS12	0.256	0.007	2.6%	6
COMS12N	0.252	0.006	2.4%	6
COMS14	0.264	0.008	3.2%	5
COMS14N	0.254	0.010	4.1%	9
COMS16	0.262	0.010	3.8%	2
COMS16N	0.245	0.007	2.7%	4
COMS18	0.250	0.004	1.5%	3
COMS18N	0.254	0.008	3.3%	2
COMS20	0.234	0.004	1.9%	11
COMS20N	0.243	0.005	2.0%	3

## IV. DISCUSSION

The goal of this work was to show that a simple activity measurement of a preloaded plaque would be useful in ensuring that the plaque used for treatment is the one called for in the treatment plan. It has been suggested^(7)^ that a single extra seed from the same lot as the seeds in a preloaded plaque be used to verify the seed strength. In our experience, there have been several occasions when at least one seed in a lot is more than 6% away from the average (our measurements on individual seeds, as well as the accompanying NIST‐traceable seed calibration certificate). Measuring a single seed in itself does not verify the relationship with the seeds in the plaque. One is still left to assume that they are from the same lot.

This verification method is based upon establishing a mean plaque calibration factor based upon the aggregate calibration factors for a given plaque type. Potential errors are identified based upon a plaque calibration factor's deviation from this mean. Additional cross‐checks are performed by comparing the plan to assay and plan to measured plaque calibration factors.

Plaque calibration factors based on this methodology may be used to verify the activity of pre‐assembled EP917 plaques to within approximately 3.3% at one standard deviation. Using the same methodology for COMS plaques, the same plaque calibration factor can be used for all COMS plaques; the average calibration factor is 0.251, with a standard deviation of 0.012 or a coefficient of variation of 4.8%. However, for more accurate results, the COMS plaque calibration factors should be calculated for each plaque type. Using this method, the COMS plaque calibration factors can be determined within an average coefficient of variation of 2.7%.

To obtain the most meaningful results, measurement technique must be consistent and reproducible. In this study, the same Styrofoam insert was used for all measurements, centering marks were drawn on the insert to facilitate reproducible positioning, and double‐sided tape was used to prevent the plaque from moving during the measurement process. In addition, the eye plaques were always positioned in the orientation shown in Fig. 1.

**Figure 3:**
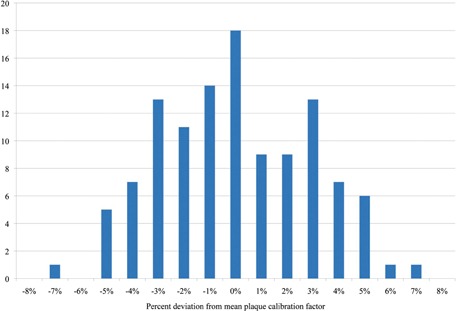
Distribution of the percent deviation from the mean plaque calibration factor for all 115 eye plaques in this study.

During this two‐year study, the air kerma strength of 115 eye plaques was checked. Of these, 11 exceeded the 5% inclusion margin. Upon further investigation, ten of these were determined to be outliers; most did not exceed the ±5% limit by more than a percent. Two plaques had deviations above ±6%: one was 6.6% and the other was −6.8%. The worst case (−6.8%) was found not to be an outlier, but rather a mismatch between the ordered seed activity and the delivered activity. Figure 3 is a histogram showing the distribution of the plaque deviations.

We have identified several possible reasons for the difference in the calibration factors among plaque types. COMS plaques have a concave shape and raised lip around the outer edge that shields laterally directed primary radiation from the ${\;}^{125}I$ seeds. The seeds in a COMS plaque are typically embedded in a Silastic carrier which is pressed into the plaque. Slight variations among the Silastic carriers, how well the seeds are seated in the carrier, and how well the carrier is seated in the plaque can cause slight variations in the laterally directed primary radiation from one plaque to another. In addition to the concave shape, the ${\;}^{125}I$ seeds in the EP917 are placed in collimating slots, which further shield laterally directed primary radiation. Slight differences in source lateral position and height within the slot can cause variations in the laterally directed primary radiation from one plaque to another.

When a suspected outlier is found, its measurements are repeated, often by another individual. The remeasured results are typically within 1% of the original measurement. Since the pre‐assembled eye plaques are not supplied with loose seeds, a secondary check of the air kerma strength of a loose seed is not possible.

## V. CONCLUSIONS

This verification method is easy to implement with commonly available equipment; all that is needed is an operable dose calibrator (with a check source) and a spreadsheet. It is effective in identifying possible errors; in our implementation an action limit is set at ±5% deviation from the mean. During this two‐year study, the air kerma strength of 115 eye plaques was checked and 11 possible errors were identified. Upon further investigation, ten of the 11 possible errors were determined to be outliers; most did not exceed the ±5% limit by more than 1%. The eleventh case was found to be a mismatch between the ordered seed activity and the delivered activity. The method described in this paper allowed us to catch the error prior to treatment. In‐house measurement of a single extra source from the batch used for the plaque would not have produced this result.

## Supporting information

Supplementary Material

Supplementary Material
